# Metagenomic Analysis of the Gut Microbiota of Wild Mice, a Newly Identified Reservoir of *Campylobacter*

**DOI:** 10.3389/fcimb.2020.596149

**Published:** 2021-02-02

**Authors:** Hyokeun Song, Junhyung Kim, Jae-Ho Guk, Woo-Hyun Kim, Hajin Nam, Jun Gyo Suh, Je Kyung Seong, Seongbeom Cho

**Affiliations:** ^1^ College of Veterinary Medicine and Research Institute for Veterinary Science, Seoul National University, Seoul, South Korea; ^2^ Department of Medical Genetics, College of Medicine, Hallym University, Chuncheon, South Korea

**Keywords:** *Campylobacter*, wild mouse, *Micromys minutus*, environmental reservoir, gut microbiota, metagenomics, *Lactobacillus*, transmission cycle

## Abstract

*Campylobacter*, the most common etiologic agent of zoonotic gastroenteritis in humans, is present in many reservoirs including livestock animals, wildlife, soil, and water. Previously, we reported a novel *Campylobacter jejuni* strain SCJK02 (MLST ST-8388) from the gut of wild mice (*Micromys minutus*) using culture-dependent methods. However, due to fastidious growth conditions and the presence of viable but non-culturable *Campylobacter* spp., it is unclear whether *M. minutus* is a *Campylobacter* reservoir. This study aimed to: 1) determine the distribution and proportion of *Campylobacter* spp. in the gut microbiota of wild mice using culture-independent methods and 2) investigate the gut microbiota of wild mice and the relationship of *Campylobacter* spp. with other gut microbes. The gut microbiota of 38 wild mice captured from perilla fields in Korea and without any clinical symptoms (18 *M. minutus* and 20 *Mus musculus*) were analyzed. Metagenomic analysis showed that 77.8% (14 of 18) of the captured *M. minutus* harbored *Campylobacter* spp. (0.24–32.92%) in the gut metagenome, whereas none of the captured *M. musculus* carried *Campylobacter* spp. in their guts. Notably, 75% (6 of 8) of *M. minutus* determined to be *Campylobacter*-negative using culture-dependent methods showed a high proportion of *Campylobacter* through metagenome analysis. The results of metagenome analysis and the absence of clinical symptoms suggest that *Campylobacter* may be a component of the normal gut flora of wild *M. minutus*. Furthermore, linear discriminant analysis (LDA) showed that *Campylobacter* was the most enriched genus in the gut microbiota of *M. minutus* (LDA score, 5.37), whereas *Lactobacillus* was the most enriched genus in *M. musculus* (LDA score, −5.96). The differences in the presence of *Campylobacter* between the two species of wild mice may be attributed to the differential abundance of *Campylobacter* and *Lactobacillus* in their respective gut microbiota. In conclusion, the results indicate that wild *M. minutus* may serve as a potential *Campylobacter* reservoir. This study presents the first metagenomics analysis of the *M. minutus* gut microbiota to explore its possible role as an environmental *Campylobacter* reservoir and provides a basis for future studies using culture-independent methods to determine the role of environmental reservoirs in *Campylobacter* transmission.

## Introduction

*Campylobacter* is one of the most common etiologic agents of zoonotic gastroenteritis in humans (Kaakoush et al., 2015). Although the most common cause of *Campylobacter* infection is the intake or handling of contaminated poultry, environmental sources such as wildlife, soil, and water are also important infection routes (Whiley et al., 2013; Hofreuter, 2014; Skarp et al., 2016). As an environmental reservoir, wildlife is an emerging source of *Campylobacter* infection *via* the direct transmission of *Campylobacter* to humans or indirectly *via* the wildlife-livestock-human cycle (Kim et al., 2020). While the majority of studies on *Campylobacter* reservoirs in wildlife have been conducted on wild birds, several studies on other hosts, such as deer, boars, and reptiles, have also been conducted (French et al., 2009; Díaz-Sánchez et al., 2013; Patrick et al., 2013; Carbonero et al., 2014). Wild mice are distributed in a wide range of habitats globally and often transmit diverse zoonotic pathogens to humans and livestock, serving as a link between wildlife and the urban community (Razzauti et al., 2015); however, *Campylobacter* in wild mice is not well understood. One study reported *Campylobacter* strains isolated from wild rodents, suggesting wild rodents as a risk factor of *Campylobacter* infection in livestock (Meerburg et al., 2006).

Most studies on *Campylobacter* in wildlife have been conducted using culture-dependent methods, such as the isolation and characterization of bacterial strain (French et al., 2009; Díaz-Sánchez et al., 2013; Patrick et al., 2013; Carbonero et al., 2014). Previously, we reported a novel *C. jejuni* strain SCJK02 (MLST ST-8388) isolated from fecal samples of wild mice (*Micromys minutus*) (Kim et al., 2020). In the previous study, *Campylobacter* was isolated from 63% of *M*. *minutus*, whereas none was isolated from *Mus musculus*. Considering the limitations of culture-dependent methods, such as fastidious growth conditions and the presence of viable but non-culturable *Campylobacter* spp. (Mihaljevic et al., 2007; Jackson et al., 2009), it is likely that *Campylobacter* was not detected, even if it was present. Therefore, it is essential to apply culture-independent methods together with traditional culture-dependent methods to precisely determine the presence of *Campylobacter* in a host.

The role of the gut microbiota in *Campylobacter*-mediated infection has been reported in several studies (Li et al., 2018; Sun et al., 2018). In humans, the microbiota of poultry workers infected with *Campylobacter* and those resistant to colonization of *Campylobacter* show significant differences in the abundance of certain genera (Dicksved et al., 2014). In laboratory mice, elevated levels of intestinal *Escherichia coli* reduce colonization resistance to *Campylobacter* (Haag et al., 2012), and the gut microbiota composition affects the extraintestinal dissemination of *Campylobacter* (O’Loughlin et al., 2015). In poultry, neonatal chickens transplanted with mature microbiota show a reduced transmission potential of *Campylobacter* (Gilroy et al., 2018). Thus, the infection risk of *Campylobacter* is affected by the gut microbiota of the host through diverse microbe-microbe interactions. Since the gut microbiota of *M. minutus* has not yet been investigated, studies are needed to improve the prediction and prevention of the transmission of *Campylobacter* from wildlife to humans.

This study was conducted to: 1) determine the distribution and proportion of *Campylobacter* spp. in the gut microbiota of wild mice using culture-independent methods and 2) investigate the core microbiota of wild mice and the relationship of *Campylobacter* spp. with other gut microbes. The gut microbiota of 38 wild mice without clinical symptoms (18 *M. minutus* and 20 *M. musculus*) and captured for 2 years from perilla fields in Korea at the end of winter torpor were analyzed. This study is the first to investigate the gut microbiota of *M. minutus* using metagenomics to explore its possible role as an environmental *Campylobacter* reservoir.

## Materials and Methods

### Study Design and Sample Collection

The Institutional Animal Care and Use Committee of Hallym University (approval number Hallym2017-5, Hallym 2018-6) approved this study. Two species of wild mice *(M. minutus* and *M. musculus*) were captured for 2 years from the perilla fields of Chuncheon in Korea at the end of their winter torpor. Information on the wild mice used in this study is included in the supplementary material (**Supplementary Table 1**). All captured mice were transferred to the lab facility immediately. Fresh fecal samples from the mice were collected in single cages and stored at −80°C.

In our previous study, *Campylobacter* was isolated from mice fecal samples using two different culture methods (Kim et al., 2020). Briefly, homogenized fecal samples (in phosphate-buffered saline—PBS) were directly spread onto modified cefoperazone–deoxycholate agar plates (mCCDA; Oxoid Ltd., Hampshire, United Kingdom) containing the CCDA-selective supplement (Oxoid, Ltd.) and plates were incubated at 42°C for 2 days under microaerobic conditions. Next, *Campylobacter*-like colonies were inoculated into Müller–Hinton agar plates (Oxoid Ltd.) and then tested by *Campylobacter* genus-specific polymerase chain reaction (PCR) (Wang et al., 2002). All *Campylobacter*-positive colonies were identified as *C. jejuni* by species-specific PCR (Wang et al., 2002). Additionally, fecal samples that were *Campylobacter-*negative subjected to enrichment in Bolton broth (Oxoid, Ltd.) containing the Bolton broth selective supplement (Oxoid, Ltd., Hampshire, United Kingdom) for 2 days at 42°C under microaerobic conditions. Thereafter, the presence of *C. jejuni* was investigated as above. Of note, results showed that *Campylobacter* was culture-positive in 63.6% of *M. minutus*, and culture-negative in all *M. musculus*.

Here, to investigate the differences in the gut microbiota of *Campylobacter* culture-positive and culture-negative *M. minutus*, 10 fecal samples from culture-positive *M. minutus*, and 8 fecal samples from culture-negative *M. minutus* were used for microbial community analysis. Additionally, to investigate the difference between the gut microbiota of the two wild mice species, 20 fecal samples from *M. musculus* (all *Campylobacter* culture-negative) were used for microbial community analysis.

### DNA Extraction and 16S rRNA Sequencing

Metagenomic DNA extraction from fecal samples was performed using the Fast DNA Soil Kit (MP Biomedicals, Santa Ana, CA, USA) according to the manufacturer’s instructions. The V3–V4 regions of the 16S rRNA gene were amplified using the following primers: 341F; 5′-TCGTCGGCAGCGTCAGATGTGTATAAGAGACAGCCTACGGGNGGCWGCAG-3′ and 805R; 5′-GTCTCGTGGGCTCGGAGATGTGTATAAGAGACAGGACTACHVGGGTATCTAATCC-3′. PicoGreen was used to pool and normalize the amplified products. All sequencing processes were performed using an Illumina MiSeq (San Diego, CA, USA) platform at Macrogen, Inc. (Seoul, Korea).

### Bioinformatics and Statistical Analyses

The bioinformatics analysis of the sequence data was performed using QIIME 2 (version 2019.10) software package (Bolyen et al., 2019) and *MicrobiomeAnalystR* in R package (Dhariwal et al., 2017). An amplicon sequence variant (ASV) table was generated by filtering, dereplicating, and denoising the raw sequence data using DADA2 (Callahan et al., 2016). A phylogenetic tree of representative sequences was generated using MAFFT (Katoh and Standley, 2013). Taxonomy assignment of the ASV table was conducted at the phylum and genus levels using a naïve Bayes classifier implemented in the q2-feature-classifier (Bokulich et al., 2018) against the SILVA database, version 132 (Quast et al., 2012). ASVs that were classified into the genus *Campylobacter* were further identified at the species-level. For downstream analysis, the sequencing data were normalized *via* rarefication to the minimum library size.

The alpha diversity of the microbial community was measured using the phyloseq package with two metrics, including the number of observed ASVs, which accounts for richness, and the Simpson’s and Shannon’s indexes, which account for richness and evenness (McMurdie and Holmes, 2013). Differences in alpha diversity between wild mice groups were evaluated using the Mann-Whitney U test. Beta diversity was measured based on Bray-Curtis dissimilarity, and the differences in beta diversity between wild mice groups were evaluated using the analysis of group similarities (ANOSIM) test. Sample core microbiota were defined as those with a minimum abundance of 0.01% and a prevalence of 50% as the cut-off values. Differential abundance analysis of microbiota was performed using linear discriminant analysis effect size (LEFSe), implemented in *MicrobiomeAnalystR* in the R package (Segata et al., 2011). We considered a *p* value lower than 0.05 to indicate significance. Statistical analyses were performed using SPSS 25 (SPSS, Inc., Chicago, IL, USA) and R version 3.6.3.

## Results

### Taxonomic Composition of the Gut Microbiota of Wild Mice

**Figure 1:**
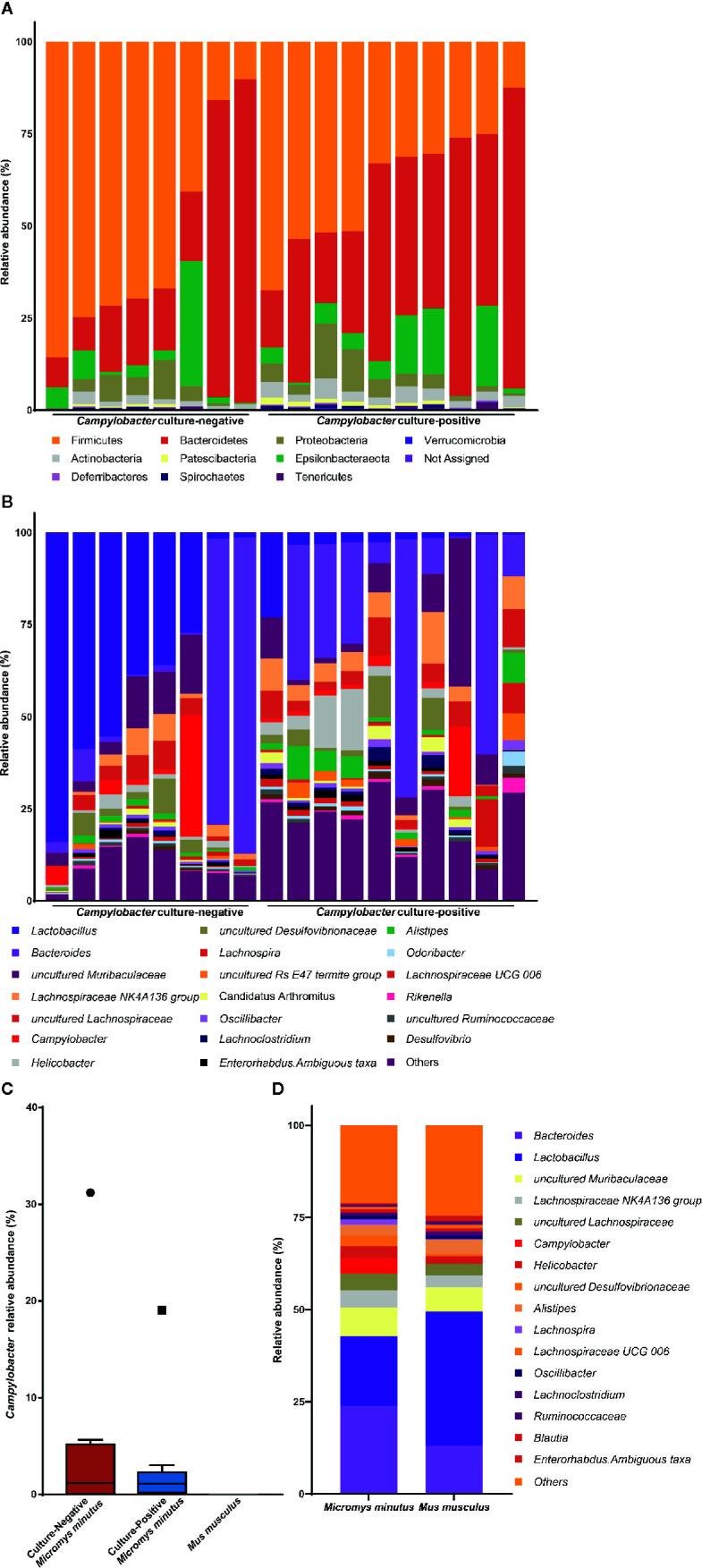
Taxonomic composition of the gut microbiota of wild mice. Taxonomy bar plot of the gut microbiota of *Micromys minutus* at the **(A)** phylum and **(B)** genus levels. **(C)** The relative abundance of *Campylobacter* in the gut microbiota of *Micromys minutus* and *Mus musculus*. The blue and orange boxes represent the relative abundance of *Campylobacter* in the *Campylobacter* culture-positive and culture-negative *M. minutus* groups. Circle (●) and square (▪) represent the maximum point of relative abundance of *Campylobacter*, respectively. **(D)** Taxonomic composition of gut microbiota of two species of wild mice (*Micromys minutus* and *Mus musculus*) at the genus level.

To determine the distribution and proportion of *Campylobacter* in the gut microbiota of wild mice, fecal microbiota from 18 *M. minutus* (10 culture-positive, 8 culture-negative) and 20 *M. musculus* (all culture-negative) were compared. No ASV was classified into the genus *Campylobacter* in the gut microbiota of *M. musculus*. The taxonomic composition of the gut microbiota of individual *M. minutus* at the phylum and genus levels are shown in **Figures 1A, B**. *Campylobacter* was present (0.24–32.92%) in the gut microbiota of 14 of 18 *M. minutus* (77.8%) but not in any of the *M. musculus*. The relative abundance of *Campylobacter* in the culture-positive and -negative groups of *M. minutus* showed no significant difference according to the Mann-Whitney U test (*p* > 0.05) (**Figure 1C**). Of note, all ASVs classified into the genus *Campylobacter* were identified as *C. jejuni* at the species-level.

The microbiota of all *M. minutus* samples comprised nine main bacterial phyla including Firmicutes, Bacteroidetes, Epsilonbacteraeota, Proteobacteria, Actinobacteria, Patescibacteria, Deferribacteres, Spirochaetes, and Tenericutes. Firmicutes (45.47%) was the most dominant phylum, followed by Bacteroidetes (38.61%) and Epsilonbacteraeota (7.34%). At the genus level, *Bacteroides* (23.79%) was the most dominant genus, followed by *Lactobacillus* (18.92%), uncultured *Muribaculaceae* (5.96%), *Lachnospiraceae NK4A136 group* (4.67%), uncultured *Lachnospiraceae* (4.65%), *Campylobacter* (4.03%), and *Helicobacter* (3.30%). The microbiota of *M. musculus* comprised seven main bacterial phyla, including Firmicutes, Bacteroidetes, Epsilonbacteraeota, Actinobacteria, Proteobacteria, Patescibacteria, and Deferribacteres. Firmicutes (62.02%) was the most dominant phyla, followed by Bacteroidetes (32.70%) and Epsilonbacteraeota (2.00%). At the genus level, *Lactobacillus* (36.44%) was the most dominant genus, followed by *Bacteroides* (12.99%), uncultured *Muribaculaceae* (5.39%), and *Alistipes* (4.17%) (**Figure 1D**). The taxonomic composition of the gut microbiota of individual *M. musculus* is shown in **Supplementary Figure 1**.

**Figure 2:**
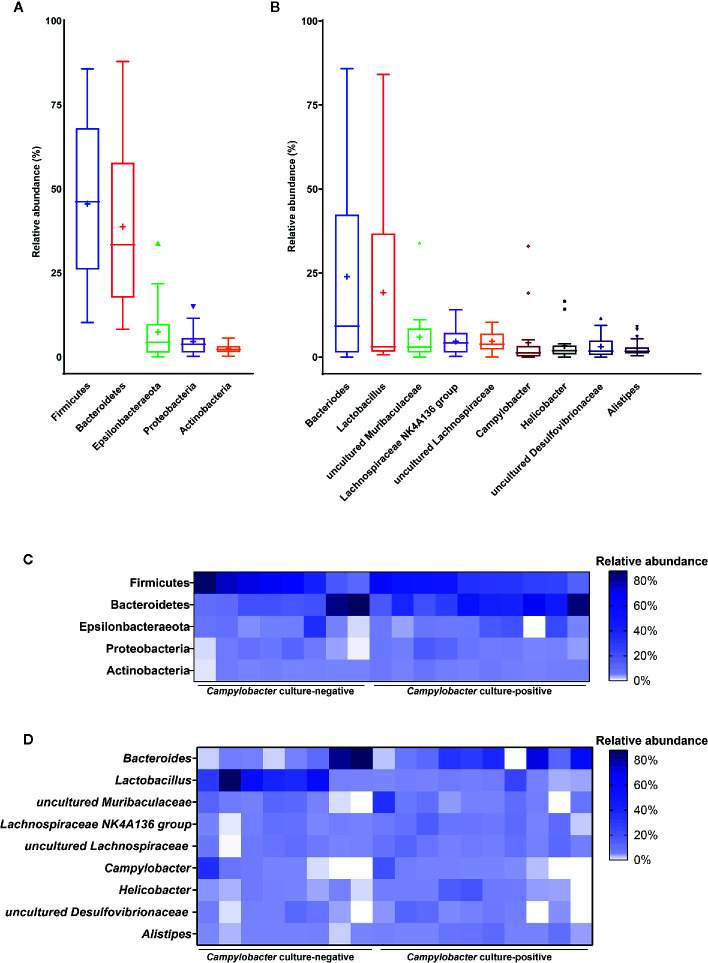
Core gut microbiota of *Micromys minutus*. Box plots showing the relative abundance of the members of the core microbiota at the **(A)** phylum and **(B)** genus levels. Plus sign (+) represents the mean value. Heatmaps showing the relative abundance of core microbiota **(C)** at the phylum and **(D)** genus levels in individual *M. minutus* samples. The X-axis represents the individual samples of *M. minutus*. The Y-axis represents the core taxa. The color scale represents the relative abundance of core taxa in individual samples.

Members of the core microbiota of *M. minutus* at the phylum level were identified as Firmicutes, Bacteroidetes, Epsilonbacteraeota, Proteobacteria, and Actinobacteria (**Figures 2A, C**). Members of the core microbiota of *M. minutus* at the genus level were identified as *Bacteroides*, *Lactobacillus*, uncultured *Muribaculaceae*, *Lachnospiraceae NK4A136 group*, uncultured *Lachnospiraceae*, *Helicobacter*, *Campylobacter*, uncultured *Desulfovibrionaceae*, and *Alistipes* (**Figures 2B, D**).

### Differences in the Gut Microbiota of *Micromys minutus* According to the Culture Results of *Campylobacter*

**Figure 3:**
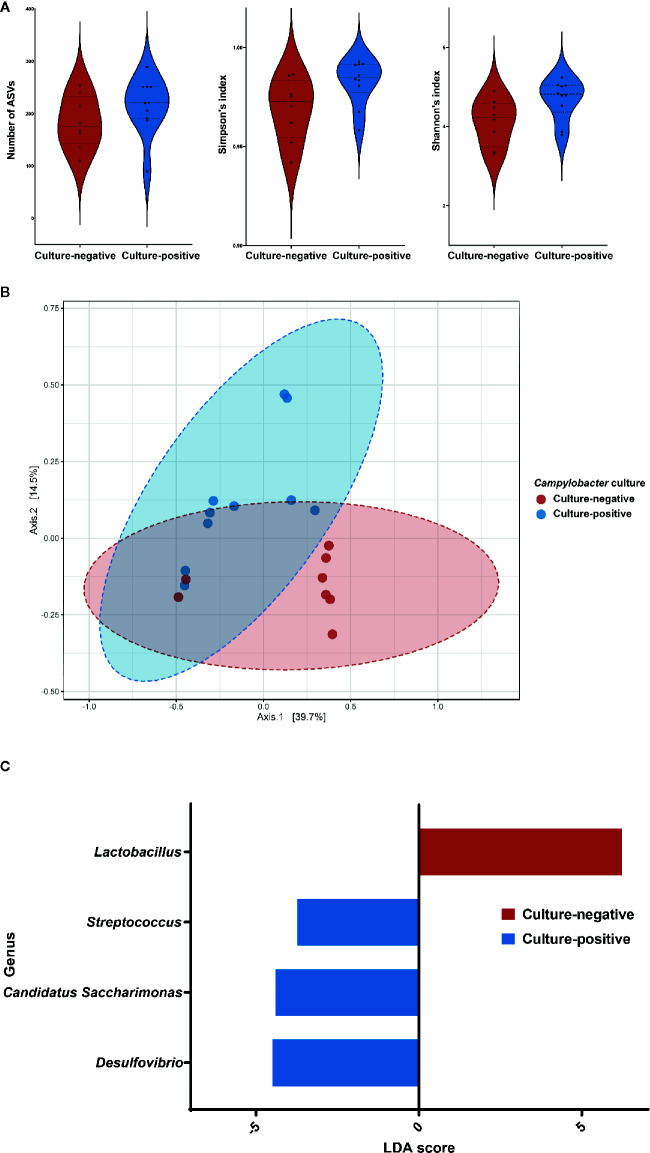
Differences in the gut microbiota of *Micromys minutus* according to *Campylobacter* culture status. **(A)** Alpha diversity of the gut microbiota of two groups of *Micromys minutus*. The distribution of the number of observed amplicon sequence variants, the Simpson’s index and the Shannon’s index of each group is shown in the box plot. The blue box denotes the *Campylobacter* culture-positive group, and the red box denotes the *Campylobacter* culture-negative group. **(B)** Principle coordinate analysis plot of Bray-Curtis dissimilarity between the gut microbiota of the *Campylobacter* culture-negative and -positive groups of *M. minutus*. Ellipses indicate 95% confidence intervals. **(C)** Histograms of the linear discriminant analysis scores for genera with differential abundance identified using linear discriminant analysis effect size in a culture-positive (blue) and culture-negative (red) group of *Micromys minutus*.

When the two culture groups of *M. minutus* were compared using the Mann-Whitney test, no significant differences (*p* > 0.05) were observed in the number of observed ASVs, the Simpson’s index and the Shannon’s index (**Figure 3A**).

The beta diversity as per the principle coordinate analysis based on Bray-Curtis dissimilarity showed distinct clustering of the gut microbiota of *M. minutus* according to the *Campylobacter* culture results (**Figure 3B**). An ANOSIM test revealed a significant difference in the gut microbiota between the *Campylobacter* culture-positive and -negative groups of *M. minutus* (R: 0.23253, *p* < 0.05). Of note, no significant differences in the beta diversity of the *M. minutus* groups were detected for other factors, such as gender and habitat (*p* > 0.05).

To identify the bacterial taxa with significantly different abundances between wild mice groups, LEFSe was performed. When the *Campylobacter* culture-positive and negative groups of *M*. *minutus* were compared at the phylum level, Actinobacteria (LDA score −4.89, *p* < 0.05) was the most enriched phylum in the microbiota of *Campylobacter* culture-positive *M*. *minutus*, followed by Patescibacteria (LDA score −4.4, *p* < 0.05). At the genus level, *Lactobacillus* (LDA score 6.23, *p* < 0.05) was the most enriched genus in the microbiota of *Campylobacter* culture-negative *M. minutus*, whereas *Desulfovibrio* (LDA score −4.5, *p* < 0.05), *Candidatus Saccharimonas* (LDA score −4.4, *p* < 0.05), and *Streptococcus* (LDA score −3.73, *p* < 0.05) were enriched in *Campylobacter* culture-positive *M. minutus* (**Figure 3C**).

### Difference in the Gut Microbiota Between Two Species of Wild Mice

**Figure 4:**
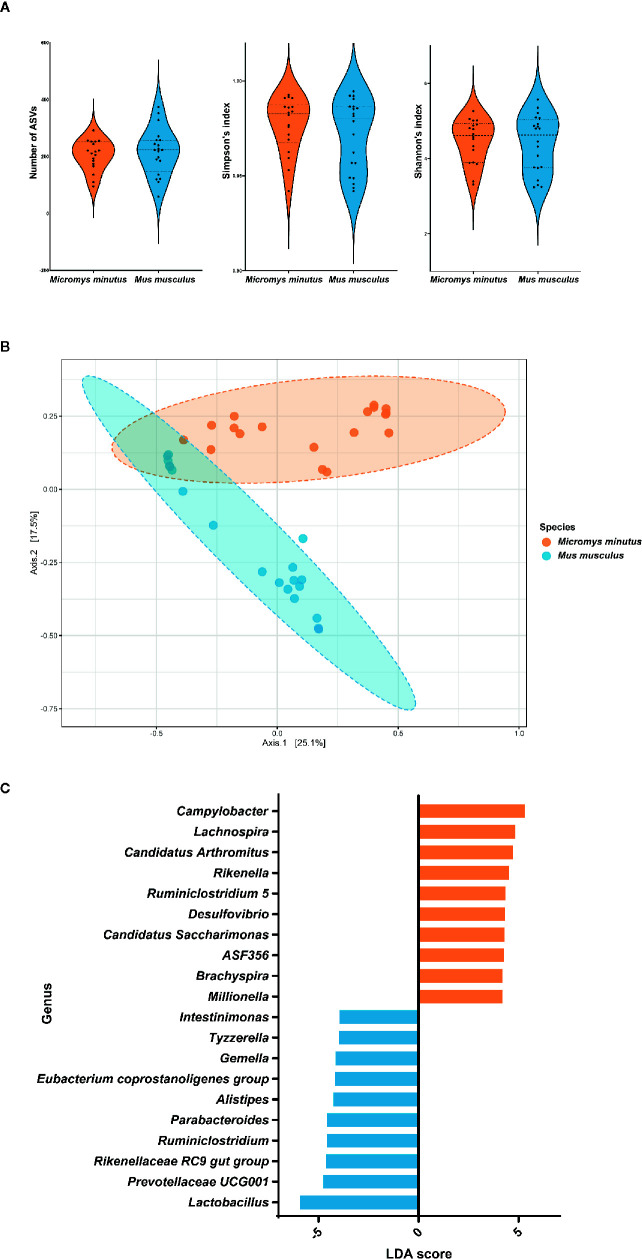
Differences in the gut microbiota of two species of wild mice. **(A)** Alpha diversity of the gut microbiota of two species of wild mice. The distribution of the number of observed amplicon sequence variants, the Simpson’s index and the Shannon’s index of each group is shown in the box plot. **(B)** Principle coordinate analysis plot of Bray-Curtis dissimilarity between the gut microbiota of *Micromys minutus* (orange) and *Mus musculus* (blue). Ellipses indicate 95% confidence intervals. **(C)** Histograms of the linear discriminant analysis scores for genera with differential abundance identified using linear discriminant analysis effect size in *M. minutus* (orange) and *M. musculus* (blue).

When the alpha diversity of two species of wild mice (*M. minutus* and *M. musculus*) was compared using the Mann-Whitney test, no significant differences (*p* > 0.05) were observed in the alpha diversity metrics, including the number of observed ASVs, the Simpson’s index, and the Shannon’s index (**Figure 4A**).

The beta diversity as per the principle coordinate analysis based on Bray-Curtis dissimilarity showed distinct clustering of the gut microbiota of wild mice according to species (**Figure 4B**). An ANOSIM test revealed a significant difference in the gut microbiota between *M. minutus* and *M. musculus* (R: 0.57627, *p* < 0.001).

When the two species of wild mice (*M*. *minutus* and *M*. *musculus*) were compared, the abundance of eight phyla, including Firmicutes, Verrucomicrobia, Deferribacteres, Spirochaetes, Patescibacteria, Actinobacteria, Proteobacteria, and Epsilonbacteraeota were found to be significantly different (*p* < 0.05) based on LEFSe. Firmicutes (LDA score −5.92) was the most enriched phylum in the gut microbiota of *M*. *musculus*, whereas Epsilonbacteraeota (LDA score 5.43) was the most enriched phylum in the gut microbiota of *M*. *minutus*, followed by Proteobacteria (LDA score 5.19), Actinobacteria (LDA score 4.69), Patescibacteria (LDA score 4.3), Spirochaetes (LDA score 4.2), Deferribacteres (LDA score 3.96), and Verrucomicrobia (LDA score 3.35). At the genus level, the abundance of all 35 genera was significantly different (*p* < 0.05). *Campylobacter* (LDA score 5.3) was the most enriched genus in *M*. *minutus*, whereas *Lactobacillus* (LDA score −5.94) was the most enriched genus in *M. musculus* (**Figure 4C**, **Supplementary Table 2**).

## Discussion

Previously, we reported a novel *C. jejuni* strain isolated from wild *M. minutus* using a culture-dependent method (Kim et al., 2020). However, the incrimination of *M*. *minutus* as a reservoir based on culture-dependent methods alone remained unclear because of difficulties in the isolation of *Campylobacter* owing to the fastidious growth conditions required (i.e., microaerophilic) and the presence of viable but non-culturable *Campylobacter* (Mihaljevic et al., 2007; Jackson et al., 2009). Moreover, numerous studies have highlighted the role of a reservoir’s microbiota composition in the transmission of a wide range of zoonotic pathogens (Jones et al., 2008; Stecher et al., 2013; Razzauti et al., 2015). However, most studies on the microbiota of wild mice have focused on that of wild *M. musculus*, belonging to the same species as the laboratory mouse, and no study has investigated the microbiota of *M. minutus* (Weldon et al., 2015; Rosshart et al., 2017; Rosshart et al., 2019). Therefore, it is essential to investigate the gut microbiota of *M*. *minutus* using a culture-independent method to predict the role of *M*. *minutus* in *Campylobacter* transmission.

The current study revealed that Firmicutes and Bacteroidetes are the most dominant phyla in the gut microbiota of *M*. *minutus*; in fact, these are the dominant phyla in a wide range of wild rodents (Debebe et al., 2017; Lavrinienko et al., 2018) and are involved in nutrition metabolism and the immune response of the host (Tremaroli and Bäckhed, 2012). Members of Firmicutes play key roles in the degradation of polysaccharides (Flint et al., 2012); thus, the high abundance of Firmicutes in the gut may be related to the food sources and habitats of *M*. *minutus* (Hata, 2011). At the genus level, *Bacteroides* and *Lactobacillus* were the predominant genera, accounting for nearly half of the microbiota composition. The high abundance of *Bacteroides* and *Lactobacillus* is consistent with the results of another study on omnivorous mammals, including wild mice (*Apodemus sylvaticus*), bears, squirrels, and lemurs (Maurice et al., 2015). The next dominant genera were uncultured *Muribaculaceae*, which is a major component of the mouse gut microbiota and a member of the family *Muribaculaceae*, which was previously known as the S24-7 group (Lagkouvardos et al., 2019), and *Lachnospiraceae NK4A136 group*, a short-chain fatty acid-producing bacteria in the gut (Hu et al., 2019). Therefore, the components of the gut microbiota of *M. minutus* appear to be comparable to those of the gut microbiota of wild rodents reported in previous studies.

Notably, *Campylobacter* was the sixth most abundant genus in the microbiota of all *M*. *minutus* and varied among samples; this high abundance is inconsistent with previous studies on the microbiota of wild mice (Maurice et al., 2015; Weldon et al., 2015; Rosshart et al., 2017; Rosshart et al., 2019). Moreover, most *M*. *minutus* harbored *Campylobacter* in their gut metagenome. Of note, this high prevalence of *Campylobacter* in the gut microbiota is similar to that in poultry, which is known to harbor *Campylobacter* as part of the normal gut flora (O’Sullivan et al., 2000; Sahin et al., 2002; Humphrey, 2006). Moreover, the concept of core microbiota considers not only the abundance but also the prevalence to identify microbial communities that exist persistently (Shade et al., 2012; Astudillo-García et al., 2017); thus, *Campylobacter* appears to be a member of the core microbiota of the gut of *M. minutus*. Furthermore, when laboratory mice are infected with *Campylobacter*, clinical signs of campylobacteriosis, such as a ruffled coat, hunched posture, lethargy, and diarrhea are observed (Stanfield et al., 1987; Mansfield et al., 2008; Liu et al., 2018). Therefore, if the high abundance and prevalence of *Campylobacter* in the gut microbiota of *M. minutus* were due to an external infection, there would have been clinical signs of campylobacteriosis in *M*. *minutus*; however, no clinical signs were observed in any captured mice. Considering the results of metagenome analysis and the absence of clinical signs, *Campylobacter* may exist as a normal component of the gut microbiota of *M. minutus*.

The core microbiota of *M*. *minutus* contained taxa that, in previous studies, were shown to be members of the microbiota of wild mice (*A. sylvaticus*) and laboratory mice, such as *Alistipes* (Maurice et al., 2015) and uncultured *Desulfovibrionaceae* (Zhang et al., 2010). Notably, *Helicobacter*, which can infect humans and other hosts (Bagheri et al., 2015; Tohidpour, 2016) is also a member of the core microbiota of *M. minutus*. Previous studies suggested wild mice (*M. musculus molossinus* and *A. sylvaticus*) as a reservoir of diverse *Helicobacter* strains according to culture-dependent (Won et al., 2002) and culture-independent methods (Maurice et al., 2015); however, the possibility of *M*. *minutus* as a potential reservoir of other zoonotic pathogens has not been studied. Future studies using culture-dependent methods for further analyses, such as the isolation and characterization of pathogens, are needed to explore the potential of wild mice as a reservoir of other zoonotic pathogens.

Metagenomic analysis results showed that most of the captured *M. minutus* harbored *Campylobacter* in the gut metagenome, regardless of their culture status. Notably, most *M. minutus* that were determined to be *Campylobacter*-negative by culture-dependent methods harbored high proportions of *Campylobacter* in the gut metagenome, indicating that culture-dependent methods alone cannot reliably indicate whether *Campylobacter* is present in the gut. This may be attributed to difficulties in the isolation of *Campylobacter* (as mentioned above) or the cultivation of *Campylobacter* may have been affected by components of the gut microbiota, such as competing flora that inhibit the growth of *Campylobacter* (Jasson et al., 2009; Hazeleger et al., 2016). Moreover, the difference in the microbiota composition between the culture-positive and -negative groups may have affected the isolation of *Campylobacter*. Beta diversity analysis, which showed that the microbiota of *M*. *minutus* was clustered by the *Campylobacter* culture results rather than by other factors such as gender or habitat, supported this possibility. Differential abundance analysis showed that *Lactobacillus* was the only significantly enriched genus in the culture-negative group compared to that in the culture-positive group. Previous studies revealed that the growth of *Campylobacter* in co-cultures of *Campylobacter* and *Lactobacillus* was significantly lower than that in a single culture of *Campylobacter*, indicating that *Lactobacillus* acts as an antagonist to reduce the level of *Campylobacter* in culture (Wang et al., 2014; Taha-Abdelaziz et al., 2019). These results support the possibility that the relatively high abundance of *Lactobacillus* in the culture-negative group affected the isolation of *Campylobacter* during the culture procedures. As studies on the characteristics of *Lactobacillus* strains isolated from wild mice are lacking, further studies are needed to better understand the antagonistic activities of wild mice-derived *Lactobacillus* strains on *Campylobacter*.

The presence of *Campylobacter* in the gut of the two species of wild mice was also very distinctly different by species. Most *M. minutus* harbored *Campylobacter* in their gut, whereas none of the *M*. *musculus* harbored *Campylobacter* in their gut. Notably, the presence of *Campylobacter* differed remarkably, despite the fact that the two species of mice were captured in adjacent areas. These results suggest that the different microbiota composition of the two species of wild mice may affect the colonization of *Campylobacter* in the gut. Recent studies showed that components of the gut microbiota provide colonization resistance to *Campylobacter* by competing for nutrition, by modulating the host immune response, and through direct antagonism (Neish, 2009; O’Loughlin et al., 2015; Kampmann et al., 2016); thus, the components of the microbiota in wild *M*. *musculus* may have prevented the colonization of *Campylobacter* in their gut. Differential abundance analysis to identify significantly enriched taxa in *M*. *musculus* showed that *Lactobacillus* was the most enriched genus in *M. musculus*. Diverse *Lactobacillus* strains are known to reduce the colonization of *Campylobacter* in the gut (Alemka et al., 2012; Sicard et al., 2017); thus, highly abundant *Lactobacillus* may have played a role as a prophylactic agent against *Campylobacter* in the gut of *M*. *musculus*. Further studies are needed to demonstrate the interaction of the gut microbiota and colonization of *Campylobacter* in wild mice.

## Conclusion

This study is the first to investigate the gut microbiota of *M. minutus* using metagenomics to explore its possible role as an environmental *Campylobacter* reservoir. This culture-independent approach indicated that wild *M. minutus* may serve as a reservoir of *Campylobacter*. Metagenomic analysis results revealed that most *M. minutus* harbored high proportions of *Campylobacter* in the gut microbiota regardless of culture status, indicating the necessity of using a culture-independent method together with traditional culture-dependent methods to precisely determine the presence of *Campylobacter*. Considering the high abundance and prevalence of *Campylobacter* in the gut microbiota, and the absence of clinical symptoms, *Campylobacter* may be a component of the normal gut flora of wild *M. minutus.* These findings provide a basis for future studies on the role of environmental reservoirs in the transmission cycle of *Campylobacter* using culture-independent methods.

## Data Availability Statement

The data sets presented in this study can be found in online repositories. The names of the repository/repositories and accession number(s) can be found below: https://www.ncbi.nlm.nih.gov/, PRJNA656071.

## Ethics Statement

The animal study was reviewed and approved by The Institutional Animal Care and Use Committee of Hallym University.

## Author Contributions

SC conceived and designed the study. HS, JK, and J-HG performed the sampling and experiments. HS, WK, and HN analyzed the data. JGS and JKS prepared and reviewed the manuscript. HS made a great contribution to the experiments, data analysis, and preparing the manuscript. All authors contributed to the article and approved the submitted version.

## Funding

This research was supported by the National Research Foundation of Korea (NRF-234 2018R1A2B6002396) and the Korea Mouse Phenotyping Project (2014M3A9D5A01075129 and 2016M3A9D5A01952417) of the Ministry of Science, ICT and Future Planning.

## Conflict of Interest

The authors declare that the research was conducted in the absence of any commercial or financial relationships that could be construed as a potential conflict of interest.
