# Supplementary material for: Metagenomic Analysis of the Gut Microbiota of Wild Mice, a Newly Identified Reservoir of Campylobacter
Source: Front Cell Infect Microbiol. 2021 Feb 2;10:596149. doi: 10.3389/fcimb.2020.596149 (PMC7884769; doi:10.3389/fcimb.2020.596149)
Supplement: Supplementary file 1 [file DataSheet_1.docx]

Supplementary Material

# Supplementary Figures and Tables

## Supplementary Figures

**Supplementary Figure 1.** **Taxonomic composition of the gut microbiota of wild *Mus musculus.*** (a) taxonomy bar plot of wild *M. musculus* at the phylum and (b) and genus levels.

## Supplementary Tables

**Supplementary Table 1.** Information of wild mice used in this study.

| Sample | Gender | Sampling Month | Sampling Year | Sampling Location | Species | *Campylobacter* culture result |
| --- | --- | --- | --- | --- | --- | --- |
| M1 | Male | 4 | 2019 | Dang-Rim | *Micromys minutus* | negative |
| M2 | Female | 4 | 2019 | Madang-kyo | *Micromys minutus* | negative |
| M3 | Male | 4 | 2019 | Madang-kyo | *Micromys minutus* | negative |
| M4 | Male | 4 | 2019 | Dongsan-ri | *Micromys minutus* | negative |
| M5 | Male | 4 | 2019 | Dongsan-ri | *Micromys minutus* | negative |
| M6 | Male | 4 | 2019 | Dongsan-ri | *Micromys minutus* | negative |
| M7 | Male | 4 | 2019 | Dongsan-ri | *Micromys minutus* | negative |
| M8 | Male | 4 | 2019 | Dongsan-ri | *Micromys minutus* | negative |
| M9 | Male | 4 | 2019 | Dang-Rim | *Micromys minutus* | positive |
| M10 | Male | 4 | 2019 | Pal-Mi-gil | *Micromys minutus* | positive |
| M11 | Male | 4 | 2019 | Pal-Mi-gil | *Micromys minutus* | positive |
| M12 | Male | 4 | 2019 | Dongsan-ri | *Micromys minutus* | positive |
| M13 | Male | 4 | 2019 | Dongsan-ri | *Micromys minutus* | positive |
| M14 | Female | 4 | 2019 | Dongsan-ri | *Micromys minutus* | positive |
| M15 | Female | 4 | 2019 | Dongsan-ri | *Micromys minutus* | positive |
| M16 | Female | 4 | 2019 | Dongsan-ri | *Micromys minutus* | positive |
| M17 | Female | 4 | 2019 | Dongsan-ri | *Micromys minutus* | positive |
| M18 | Female | 4 | 2019 | Dongsan-ri | *Micromys minutus* | positive |
| MM1 | Male | 4 | 2017 | Gunja-ri | *Mus musculus* | negative |
| MM2 | Female | 4 | 2017 | Gunja-ri | *Mus musculus* | negative |
| MM3 | Male | 4 | 2017 | Gunja-ri | *Mus musculus* | negative |
| MM4 | Male | 4 | 2017 | Gunja-ri | *Mus musculus* | negative |
| MM5 | Female | 3 | 2017 | Dongchon-ro | *Mus musculus* | negative |
| MM6 | Female | 3 | 2017 | Gunja-ri | *Mus musculus* | negative |
| MM7 | Baby | 4 | 2017 | Gunja-ri | *Mus musculus* | negative |
| MM8 | Baby | 4 | 2017 | Gunja-ri | *Mus musculus* | negative |
| MM9 | Baby | 4 | 2017 | Gunja-ri | *Mus musculus* | negative |
| MM10 | Baby | 4 | 2017 | Gunja-ri | *Mus musculus* | negative |
| MM11 | Male | 4 | 2017 | Yupo-ri | *Mus musculus* | negative |
| MM12 | Female | 4 | 2017 | Yupo-ri | *Mus musculus* | negative |
| MM13 | Female | 4 | 2017 | Boksa | *Mus musculus* | negative |
| MM14 | Male | 4 | 2017 | Boksa | *Mus musculus* | negative |
| MM15 | Male | 4 | 2017 | Yupo-ri | *Mus musculus* | negative |
| MM16 | Female | 4 | 2017 | Yupo-ri | *Mus musculus* | negative |
| MM17 | Male | 4 | 2017 | Joyang | *Mus musculus* | negative |
| MM18 | Female | 4 | 2017 | Joyang | *Mus musculus* | negative |
| MM19 | Male | 4 | 2017 | Boksa | *Mus musculus* | negative |
| MM20 | Female | 4 | 2017 | Boksa | *Mus musculus* | negative |

**Supplementary Table 2.** Genera showing significant difference between wild *Micromys minutus* and *Mus musculus* in LEfSe analysis

| Genus | p value | LDA score |
| --- | --- | --- |
| *Campylobacter* | 2.23E-06 | 5.3 |
| *Lachnospira* | 0.01192 | 4.83 |
| *Candidatus Arthromitus* | 1.64E-05 | 4.72 |
| *Rikenella* | 7.01E-06 | 4.53 |
| *Ruminiclostridium 5* | 0.00342 | 4.35 |
| *Desulfovibrio* | 0.019884 | 4.32 |
| *Candidatus Saccharimonas* | 0.013334 | 4.3 |
| *ASF356* | 3.77E-05 | 4.26 |
| *Millionella* | 6.65E-07 | 4.2 |
| *Brachyspira* | 0.000161 | 4.2 |
| *Lachnospiraceae UCG 001* | 0.005724 | 4.11 |
| *Eubacterium brachy group* | 0.00606 | 4.11 |
| *Mycoplasma* | 0.000161 | 4.06 |
| *Odoribacter* | 0.000475 | 3.98 |
| *Mucispirillum* | 0.009778 | 3.96 |
| *Ruminiclostridium 6* | 0.000161 | 3.95 |
| *Anaerotruncus* | 0.044704 | 3.95 |
| *GCA 900066575;Ambiguous taxa* | 0.001033 | 3.89 |
| *Butyricicoccus* | 0.037967 | 3.43 |
| *Candidatus Stoquefichus* | 0.028165 | 2.95 |
| *Anaeroplasma* | 0.048213 | -3.21 |
| *Ruminococcaceae UCG 010* | 0.012864 | -3.37 |
| *Escherichia Shigella* | 0.04817 | -3.37 |
| *Ruminococcaceae UCG 013* | 0.048213 | -3.39 |
| *Angelakisella* | 0.04817 | -3.5 |
| *Intestinimonas* | 0.002221 | -3.96 |
| *Tyzzerella* | 0.00186 | -3.98 |
| *Gemella* | 5.25E-05 | -4.15 |
| *Eubacterium coprostanoligenes group* | 0.000302 | -4.18 |
| *Alistipes* | 2.05E-05 | -4.26 |
| *Ruminiclostridium* | 0.009903 | -4.59 |
| *Parabacteroides* | 0.014464 | -4.59 |
| *Rikenellaceae RC9 gut group* | 6.65E-05 | -4.61 |
| *Prevotellaceae UCG 001* | 0.003149 | -4.76 |
| *Lactobacillus* | 0.017882 | -5.94 |
