# Supplementary figures and images for: Metagenomic Analysis of the Gut Microbiota of Wild Mice, a Newly Identified Reservoir of Campylobacter
Source: Front Cell Infect Microbiol. 2021 Feb 2;10:596149. doi: 10.3389/fcimb.2020.596149 (PMC7884769; doi:10.3389/fcimb.2020.596149)

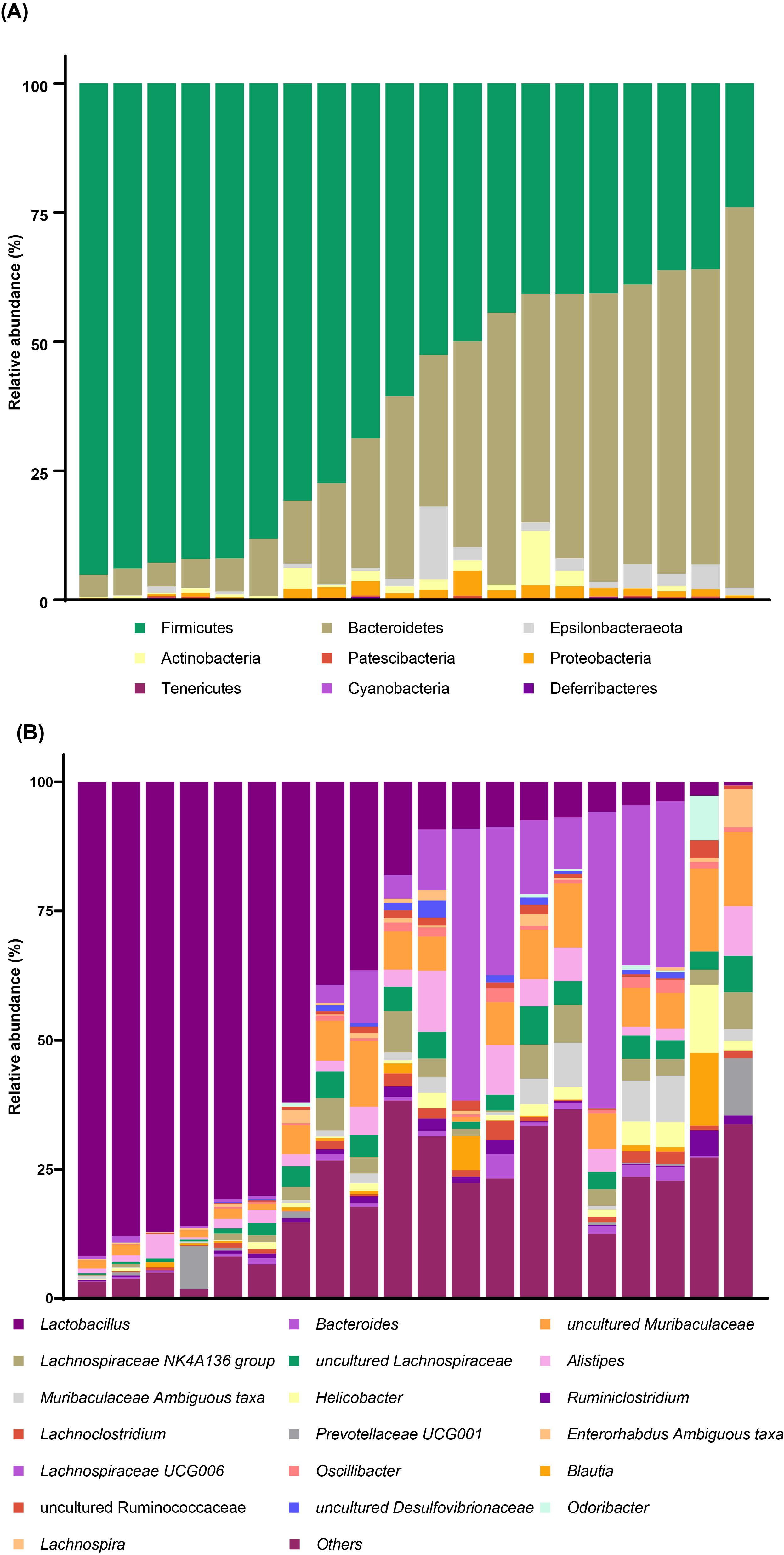

Supplement: Supplementary file 2 [file Image_1.tif]
